# Differences of bleedings after percutaneous coronary intervention using femoral closure and radial compression devices

**DOI:** 10.1097/MD.0000000000015501

**Published:** 2019-05-17

**Authors:** Seung-Hyun Kim, Michael Behnes, Sebastian Baron, Tetyana Shchetynska-Marinova, Melike Tekinsoy, Kambis Mashayekhi, Ursula Hoffmann, Martin Borggrefe, Ibrahim Akin

**Affiliations:** a First Department of Medicine, University Medical Centre Mannheim (UMM), Faculty of Medicine Mannheim, University of Heidelberg, European Center for AngioScience (ECAS), and DZHK (German Center for Cardiovascular Research) partner site Heidelberg/Mannheim, Mannheim, Germany; b Division of Cardiology and Angiology II, University Heart Center Freiburg - Bad Krozingen, Bad Krozingen, Germany.

**Keywords:** bleeding, femoral closure device, MACE, PCI, radial compression devices

## Abstract

Bleedings represent most relevant complications being correlated with significant rates of adverse clinical outcomes in patients undergoing percutaneous coronary intervention (PCI). To reduce bleeding and improve prognosis various types of vascular closure devices (VCD) are frequently applied. This study aims to compare directly one specific femoral closure (FC) to one specific radial compression (RC) device in patients after PCI focusing on overall and access-site bleedings as well as major adverse cardiac events (MACE).

This single-center, prospective, and observational study included consecutive patients either treated by the FC (StarClose SE) or RC (TR Band) device following PCI. The primary outcome was bleeding; the secondary outcomes were MACE at 30 days of follow-up.

Two hundred patients in each group were enrolled following PCI. Access-site bleeding was significantly higher in the FC (43%) compared to the RC (30%) group (*P* = .001). Most common type of access-site bleeding consisted of hematomas. Of these, small and large hematomas were significantly higher in the FC group (*P* < .05). No significant differences of MACE were observed in both groups. In multivariable logistic regression models no consistent significant association of any risk factor with bleeding complications was identified.

Despite the use of VCD, transfemoral arterial access is still associated with a higher rates of access site bleeding consisting mostly of hematomas compared to trans-radial access, whereas no differences of MACE were observed between FC and RC patients at 30 days follow-up.

## 1 Introduction

Bleedings following percutaneous coronary intervention (PCI) represent one of the most relevant complications being significantly associated with an increased short- and long-term mortality in patients undergoing PCI.^[1,2]^ A meta-analysis of three randomized controlled studies (OASIS, OASIS-2, and CURE) revealed an increased incidence of death during the first 30 days in patients with major bleeding compared to those without (12.8% vs 2.5%).^[3]^ Furthermore, an increased 1-year long-term mortality in patients with major bleeding was demonstrated in a pooled analysis including 17,034 patients from three large randomized trials (REPLACE-2, ACUITY, and HORIZONS-AMI).^[4]^

Over the past several years, multidisciplinary approaches with improved medical therapy and innovative interventional devices have been made to reduce bleeding risks.^[5–7]^ The meta-analysis of 9 randomized clinical trials revealed that anticoagulation with bivalirudin might reduce major and minor bleeding risks compared with heparin plus glycoprotein IIb/IIIa inhibitors in patients undergoing PCI.^[8]^ At the same time, utilization of vascular closure devices (VCD) and smaller sheath size and cardiac catheters with better trackability as innovative interventional devices might contribute to reducing the risk of bleeding.^[9]^ Moreover, a more frequent use of trans-radial access (TRA) could lead to a reduction of major bleeding and major adverse cardiac events (MACE).^[10]^ In comparison to transfemoral access (TFA) TRA was shown to decrease significantly procedure related bleeding because of an easier application of external manual compression following PCI.^[11]^

In the case of TFA, besides manual compression and application of pressure bandages around the hips the above mentioned VCD were developed in the early 90s to reduce access site bleeding.^[12]^ VCD being collagen, suture, or clip based are used to decrease access site bleeding and to reduce post interventional time to hemostasis.^[13,14]^ Moreover, application of VCD revealed advantages of patients early mobilization and rehabilitation.^[15]^

However, the efficiencies and direct comparisons of these devices in real-life settings have been rarely investigated. Therefore, this study aims to compare directly one specific vascular femoral closure (FC) device (StarClose SE, Abbott, IL) with one specific radial compression (RC) device (TR Band, Terumo Corporation, Tokyo, Japan) in patients after PCI focusing on overall and access site bleedings as well as MACE at short-term follow-up.

## 2 Methods

### 2.1 Study population

The present study was conducted as a single-center prospective, nonrandomized study being performed at the First Department of Medicine, University Medical Centre Mannheim (UMM) in Mannheim, Germany. The study was designed as an open-label; observational all-comers study in order to recruit a generalizable and representative study population comparable to the daily practice in other PCI centers. The study was carried out according to the principles of the Declaration of Helsinki and was approved by the medical ethics commission II of the Medical Faculty Mannheim, University of Heidelberg, Germany. Written informed consent is obtained from all participating patients or their legal representatives.

Patients being planned for PCI were screened at our cardiologic department and included consecutively to this study, when they were treated either using radial arterial access site in combination with one specific vascular compression device (TR Band, Terumo Corporation, Tokyo, Japan) or using femoral arterial access site in combination with one specific VCD (StarClose SE, Abbott, IL). Only right not left radial access was used in this study. Patients being treated with other VSD than TR Band or StarClose SE after PCI were excluded. Patients with unsuccessful placement of the StarClose SE device immediately after PCI in the catherization laboratory were excluded. Further inclusion and exclusion criteria accorded to criteria of “The Femoral Closure versus Radial Compression Devices Related to Percutaneous Coronary Interventions” (FERARI, clinicaltrials.gov identifier: NCT02455661) study being outlined in detail in the previously published method paper.^[16]^ According to an estimation of the power using the data of the first 100 patients, a sample size of 200 patients in each group was necessary to power the study sufficiently for the primary endpoint. Therefore, 200 consecutive patients were recruited in both groups.^[16]^

### 2.2 Procedure

The interventional cardiologists involved in the study had experience of at least 300 trans-radial procedures per year each. Conduction of PCI procedure (i.e., choice of access site, sheath diameter, used technique, and PCI materials) was not influenced by the study protocol and based on the operator's discretion. Procedures with switching of access site were excluded. Heparin was used to achieve an activated clotting time (ACT) of 250–300 seconds during PCI and ACT was measured frequently for both arterial access sites. Peri-interventional additional antithrombotic treatment (i.e., bivalirudin or abciximab) as well as postinterventional loading with antiplatelet therapy was carried out according to European guidelines.^[17]^

The TR Band is used according to the product specific instructions for 4 hours as the only RC device in this study. Initially, 15 mL of air were inflated and patent hemostasis was achieved as described by Pancholy et al^[18]^ After four hours of radial compression, the TR Band is removed after gradual deflation by 2–3 mL every 30 minutes until final hemostasis. During the process peripheral perfusion, motor function, and sensibility were regularly checked. In all patients with TRA before and after PCI the radial perfusion and occlusion were investigated clinically by Allen's test without the use of pulse oximetry. For the Allen's test the patient was asked to clench his fist for about 30 seconds. And pressure was applied over the ulnar and the radial arteries so as to occlude both of them. The patient then opened the hand. It should appear blanched (pallor may be observed at the finger nails). Radial pressure was released while ulnar pressure is maintained, and the color of hand should return within 5 to 15 seconds. Post PCI radial occlusion was tested clinically by palpation of radial and ulnar pulses and the Allen test was re-applied thereafter.

In the other patients, FC was performed using the StarClose SE according to the product specific instructions applied by interventional cardiologists with experience with the StarClose SE device in at least 50 prior patients. The StarClose SE contains an introducer sheath, dilator, guidewire, and clip applier with a star shaped nitinol clip. When the primary procedure is completed, the catheter is removed and the sheath is left in place or exchanged for a StarClose SE compatible sheath. The clip applier is attached to the introducer sheath, signaled by a loud click to the operator. A button on the device is depressed to expand the flexible wings in the artery and provide the user a tactile signal of being against the anterior femoral artery. The device is applied with light traction against the arteriotomy, then a “no tension” position while stabilizing the device is assumed. A sliding element on the body of the device is then advanced, splitting the sheath as the clip is advanced to the arteriotomy. The operator is signaled the completion of the sheath splitting by another loud click. While pressing down with the device, a trigger button is depressed to deploy the clip. Subsequently, the clip applier and introducer sheath are withdrawn. The nitinol clip provides a secure extravascular closure that does not invade the vessel lumen.^[19]^

### 2.3 Data acquisition

Laboratory values (i.e., creatinine, hemoglobin, platelet count, and international normalized ratio [INR]) as well as baseline characteristics and past medical history including chronic kidney failure (glomerular filtration rate < 60 mL/min) or liver disease and heart failure (according to left ventricular ejection fraction) were collected from the in-hospital documentation system. All patients were followed up during hospital stay and until 30 days after the index procedure directly and by standardized telephone visits.

### 2.4 Definition of study outcomes

The primary outcome was defined by all relevant access site and nonaccess site bleedings within 30 days following PCI. Overall bleeding was classified according to established criteria such as the “Bleeding Academic Research Consortium” (BARC), “The Thrombolysis in Myocardial Infarction” (TIMI), and “The Global Use of Strategies to Open Occluded Arteries” (GUSTO).^[20–22]^ Access site complications were defined as hematomas, active bleedings, dissections, pseudoaneurysms, arteriovenous fistulae, and retroperitoneal hematomas.^[23]^ Access site bleedings were classified according to the FERARI classification.^[16]^

The secondary outcome consisted of MACE within 30 days of follow-up, which comprised all-cause and cardiovascular death, myocardial infarction, stent thrombosis, target lesion revascularization (TLR) as well as target vessel revascularization (TVR).

### 2.5 Statistical analysis

Statistical analysis was performed using SPSS Statistics (IBM, Armonk, NY) and GraphPad Prism (GraphPad Software, Inc., La Jolla, CA). Data are presented as medians with interquartile ranges (25th–75th percentiles) or as total numbers with group-related percentages. The *P*-values < .05 were considered statistically significant, *P*-values < .01 were considered as a statistical trend. Normal distribution of data was tested with the Kolmogorov–Smirnov test. For data with normal distribution, the Student *t* test was applied. Categorical variables were compared using the Chi-squared test, in case of low event rates the Fischer's exact test was applied. Baseline characteristics, which were shown to differ significantly between the two groups, were adjusted using uni- and multivariate logistic regression analyses for the predefined study endpoints.

## 3 Results

### 3.1 Baseline characteristics

A total of 400 patients following PCI was included in the present study. Two hundred patients were treated with the RC device and 200 patients were treated with the FC device after PCI. Mostly, baseline characteristics were distributed evenly between the RC and FC group (Table 1). TFA was significantly more often performed in patients with ST-segment elevation myocardial infarction (STEMI) (*P* = .0001) or angiographic control (*P* = .001), whereas RC was more often used in patients with stable angina pectoris (AP) (*P* = .001) or positive viability testing (*P* = .001). Patients in the RC group suffered more often from peripheral vascular disease. Patients being treated with RC revealed significantly shorter hospital stay (3.5 days with IQR [2.0–8.0], *P* = .001) compared to those with FC (7 days with IQR [4–9], *P* = .001). Radial occlusion post PCI was not present in any patient. No significant difference of preexisting antiplatelet or anticoagulation therapy before PCI between both groups was observed except for acetylsalicylic acid (ASA) (146 patients in FC group and 118 patients in RC group, *P* = .003) (Table 2). STEMI, stable AP, sheath size, preexisting antiplatelet treatment before PCI with ASA, mono loading following PCI with ASA or ticagrelor, and dual loading after PCI with ASA plus clopidogrel or ASA plus prasugrel as well as the number of thrombocytes were identified as significantly differing risk factors for bleeding complication amongst baseline characteristics (*P* < .05) in univariate group comparisons.

**Table 1:**
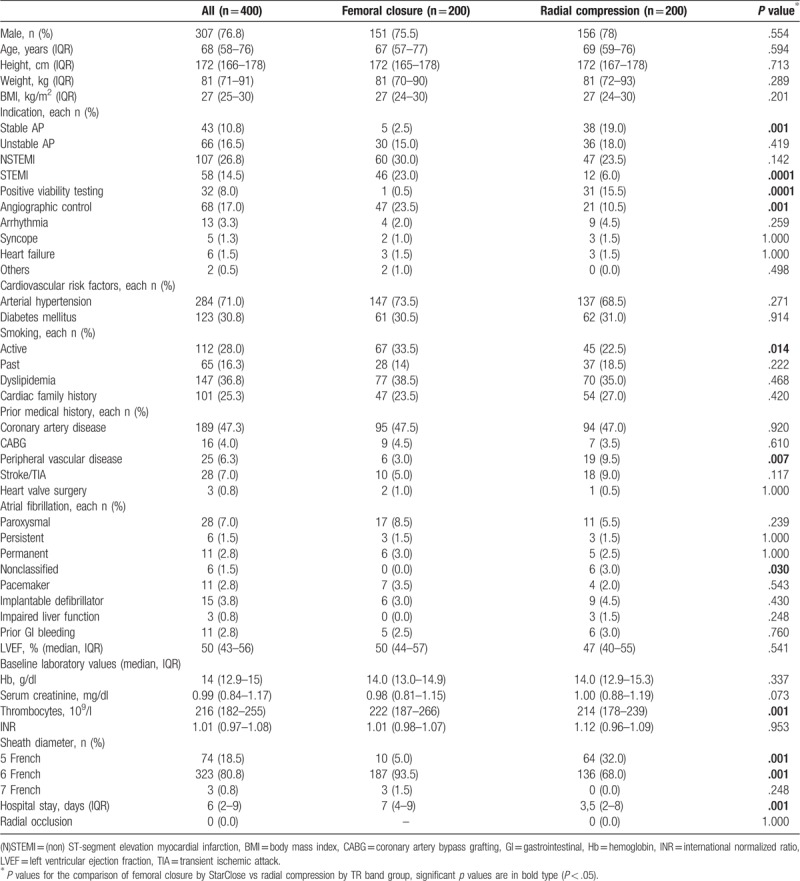
Baseline characteristics of PCI patients with application of vascular closure devices.

**Table 2:**
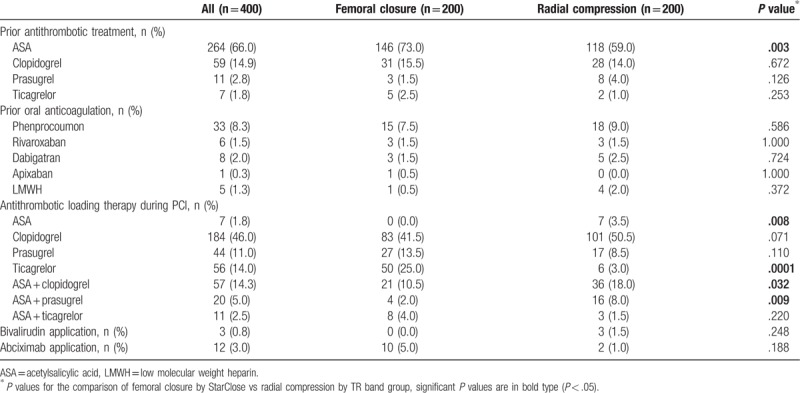
Antithrombotic therapies being used in the study.

### 3.2 Primary outcomes: bleedings within 30 days following PCI

As shown in Table 3 bleedings are classified according to BARC, TIMI, and GUSTO as well as FERARI. Due to bleeding events consisting mainly of minor hematomas, BARC type 1 constituted the majority of bleeding. BARC type 4 was not present in our study cohort because it is directly linked to coronary artery bypass grafting (CABG) surgery. For a similar reason, “minimal” in TIMI classification applied for 88% of bleeding events and only “mild” subgroup of GUSTO classification was existent. Hereby four complicated bleedings according to FERARI classification were shown. One of these was femoral artery dissection and the others were active bleedings.

**Table 3:**
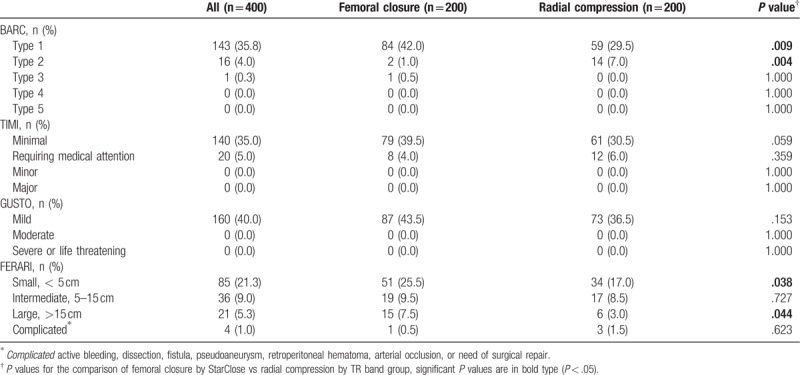
Comparison of bleedings according to bleeding classification systems in the study.

The clinical indications for PCI in this study differed significantly between TFA and TRA groups (Table 1). Table 4 presents bleedings stratified by type of procedure, that is, acute PCI for STEMI and NSTEMI, planned PCI for stable AP, unstable AP, etc, and diagnostic catheterization for angiographical control. No significant difference in a prevalence of bleedings was shown between FC and RC groups depending on type of procedure except for a small hematoma according to FERARI classification after acute PCI in patients with STEMI and NSTEMI (*P* = .003).

**Table 4:**
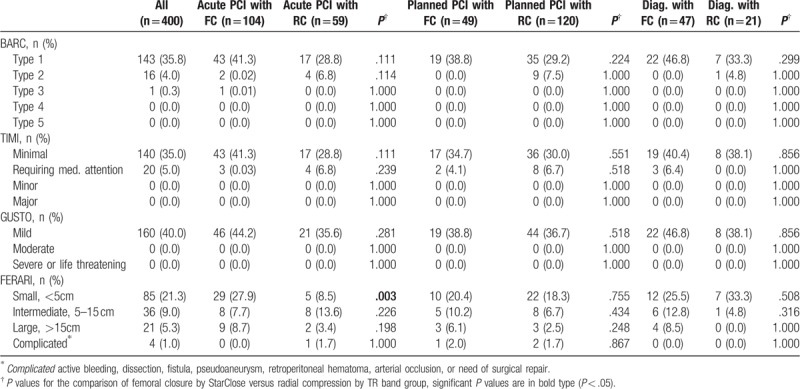
Comparison of bleedings stratified by type of procedure according to bleeding classification systems in the study.

Overall bleedings did not significantly differ between FC and RC groups (*P* = .153), whereas the prevalence of non-access site bleeding such as epistaxis, gum bleeding, and gastrointestinal bleeding was significantly higher in the RC group (*P* = .001) (Table 5). The significantly higher rate of nonaccess site bleeding in the RC group was shown to be related with significant increasing of BARC Type 2 bleeding in this group (*P* = .004). Contrastively, hematoma comprising 95% of procedure related complications was significantly increased in the FC group (*P* = .001). Subsequently, access site bleeding was categorized according to the study specific FERARI classification. Significantly increasing small and large bleeding complications according to this category were revealed in the FC group (*P* = .038, *P* = .044). However, no significant difference of intermediate or complicated bleeding events between the FC and RC group was observed.

**Table 5:**
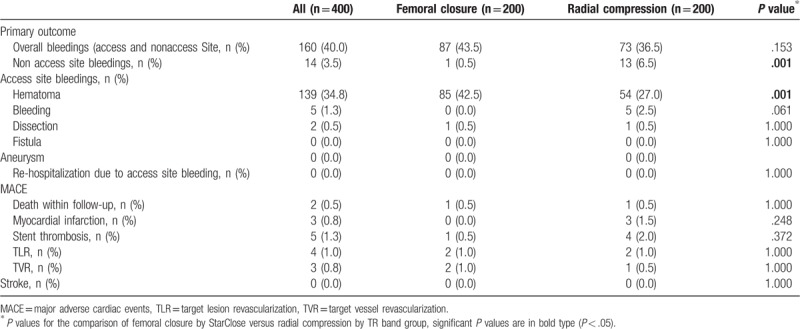
Primary and secondary outcomes in the study.

### 3.3 Secondary outcomes: MACE within 30 days following PCI

In this study MACE occurred rarely and did not differ significantly between both groups (Table 5). None of the two deaths, which occurred within 30 days of follow-up, was related to any bleeding complication. In addition, no differences of TVR and TLR were observed in both groups.

### 3.4 Multivariate logistic regression analyses for primary outcomes

Except for dual loading with ASA plus clopidogrel after PCI, none of the above described univariate significant risk factors had consistent impact on the primary outcomes in multivariate logistic regression models (Tables 6 and 7). The odds ratio of FERARI large bleeding was significantly higher in the FC group with dual loading with ASA and clopidogrel after PCI both in univariate and in multivariate analysis (odds ratio [OR] 3.594, *P* = .045 in univariate analysis; OR 3,750, *P* = .039 in multivariate analysis). It also turned out in multivariable regression model, that odds ratios of an access site bleeding and BARC Type I bleeding were significantly low in the RC group with stable AP (OR 0.308, *P* = .045; OR 0.140, *P* = .010). Moreover, dual loading with ASA plus prasugrel after PCI reduced a rate of access site hematoma in RC group (OR 0.123, *P* = .047).

**Table 6:**
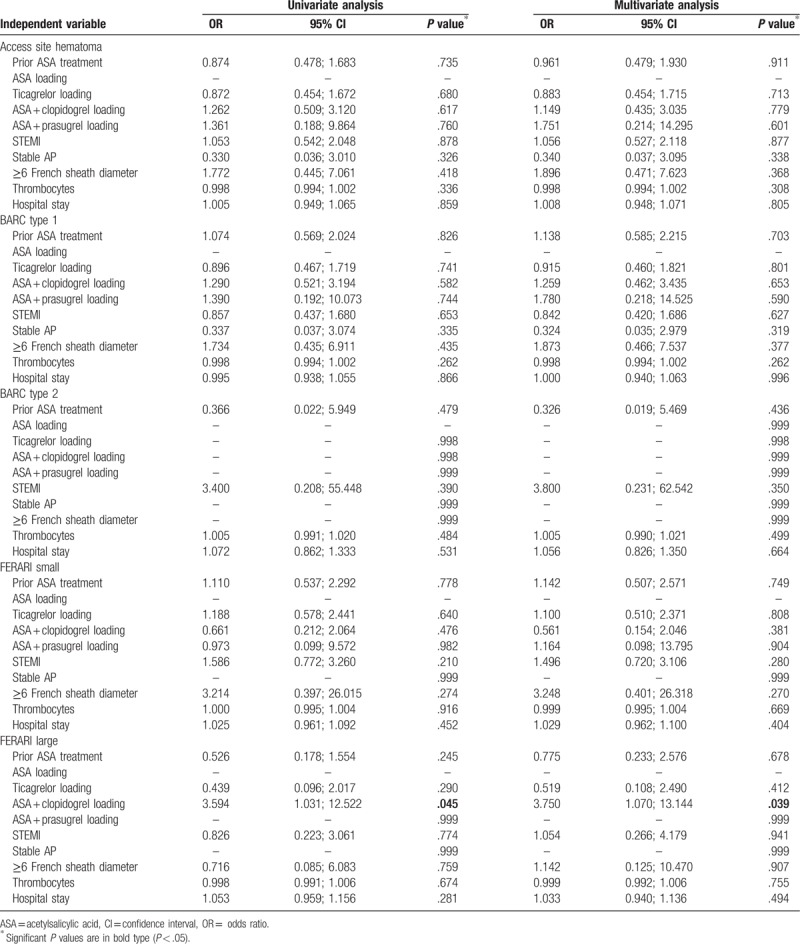
Uni- and multivariate logistic regression analyses for primary outcomes in the femoral closure group.

**Table 7:**
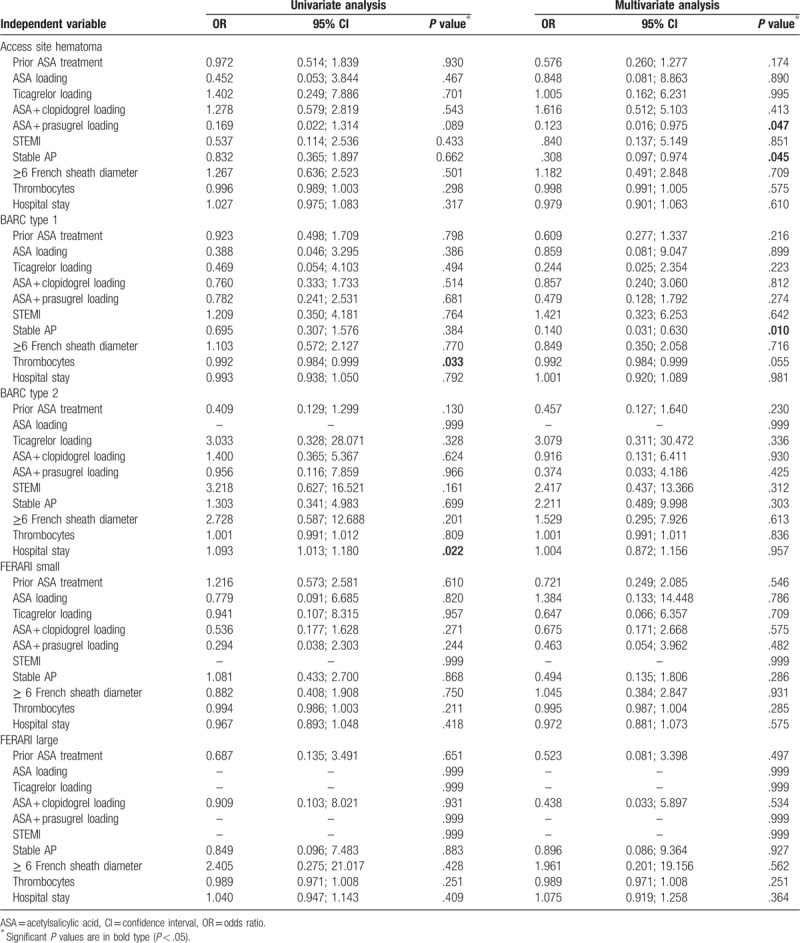
Uni- and multivariate logistic regression analyses for primary outcomes in the radial compression group.

It was noteworthy that differences of sheath diameters might not influence procedure related complications.

## 4 Discussion

This study evaluated the efficiency and comparison of VCD, especially TR Band for RC and StarClose SE for FC following PCI in real life settings. It was demonstrated that TRA with subsequent use of the TR Band for RC is significantly associated with lower rates of access site bleeding compared to TFA with application of StarClose SE for FC after PCI. A significantly higher rate of small and large hematomas as the main part of access site bleeding complications was revealed in the FC group. However, further types of bleeding, that is, active bleeding and dissection did not differ significantly between both groups. Procedure related bleedings were not influenced by differences of sheath diameters, whereas influence of number of thrombocytes on primary outcomes in the RC group might not be excluded.

Bleedings after PCI may be found at several sites, such as the arterial access site or nonaccess site, for example, intracranial, or gastrointestinal tract. However, it is still disputed whether adverse prognosis is associated with procedure related or nonprocedure related bleeding. For instance, a recent meta-analysis of 25 relevant studies demonstrated a higher adjusted risk of non-access bleeding (hazard ration [HR] 4.06, 95% confidence interval [CI]: 3.21–5.14, *P* < .00001) following PCI.^[24]^ Contrastively, another meta-analysis of three studies revealed increasing risk of mortality accompanied by similar rates of access-site and nonaccess site bleeding.^[25]^ Furthermore, severe bleedings assessed by GUSTO classification were shown to be associated with an increased risk of mortality or myocardial infarction at 6 months regardless of bleeding's origin.^[21]^

In the last two decades, besides widespread manual compression and sequential application of pressure bandages, VCD were developed continuously to improve efficiency of hemostasis following PCI, especially in the case of TFA.^[12]^ Numerous prior trials demonstrated that the application of VCD being based on collagen plug, clip, or suture-based mechanisms might significantly decrease femoral access-site bleedings in patients undergoing diagnostic cardiac catheterization and PCI compared to manual compression.^[26]^ In a nationally representative observational study by Tavris et al especially the StarClose SE VCD revealed significantly lower bleeding rates than manual compression (OR 0.77, 95% CI: 0.72–0.82).^[27]^ Additionally, in the case of RC, the TR Band for RC was shown to reduce complication rates after PCI with TRA due to its optimal hemostasis.^[28]^

Notwithstanding advantages of femoral VCD after transfemoral PCI, various prior studies comparing TFA to TRA indicated that TRA still reduces more efficiently procedure related bleedings and improve consequentially prognosis compared to TFA independently of application of VCD. Mamas et al demonstrated the independent correlation of TRA with a significantly reduced access site bleeding rates and 30-day mortality compared to TFA without using VCD in patients with baseline peri-procedure bleeding risk.^[29]^ Consequentially, it was revealed that patients at the highest risk of bleedings received the most benefit from using TRA during PCI. The RIVAL (“Radial versus femoral access for coronary angiography and intervention in patients with acute coronary syndromes”) trial indicated a significantly lower rate of access-site vascular complications in patients undergoing PCI with TRA compared to those with TFA without application of VCD.^[30]^ Rashid et al in their recent study demonstrated that TRA was associated with significantly reduced odds of bleedings (OR 0.45, CI: 0.31–0.66, *P* < .001), in-hospital mortality (OR 0.59, 95% CI 0.42–0.83, *P* = .002), MACE (OR 0.72, 95% CI: 0.55–0.94, *P* = .01), and 30-day mortality (OR 0.72, 95% CI: 0.55–0.94, *P* = .01) compared to TFA without using VCD in patients with STEMI.^[31]^

In the case of using femoral VCD after transfemoral PCI, a recently published meta-analysis revealed a significant reduction of procedure-related vascular complications (OR 0.24, 95% CI: 0.19–0.30, *P* < .001) and MACE (OR 0.88, 95% CI: 0.81–0.95, *P* = .001) in the RC group compared to the FC group.^[32]^ Sciahbasi et al demonstrated that TRA was associated with a significant reduction in major vascular complications compared to TFA even if two different VCD (AngioSeal [Terumo Coperation, Tokyo, Japan] and StarClose SE) were applied.^[33]^ Teblick et al demonstrated also that TRA was significantly associated with a lower prevalence of vascular complications compared to TFA with application of VCD (AngioSeal).^[34]^ However, in this study no significant difference of mortality rates could have been indicated. Interestingly, in contrast to prior trials, Chodor et al compared RC using TR Band versus FC using StarClose SE following PCI in patients with STEMI and showed no significant difference of access-site bleeding rates between both groups.^[35]^

Despite the use of both VCD the rates of access-site bleeding appeared to be higher than expected in the FERARI study. Access-site bleeding was shown in about 45% of patients in the FC group and 30% of patients in the RC group. The higher prevalence of procedure-related bleedings may be explained by detailed discrimination of minor bleedings according to the FERARI classification. In contrast to other classification systems FERARI categorizes more concretely hematomas being accounted for majority of access site bleedings and reported also about hematomas smaller than 5 cm (21.3%). These small hematomas contributed to low-graded bleeding of predefined other classification systems, that is, BARC type 1, TIMI minimal, and GUSTO mild.

Many previous studies assessed a significant association of major bleedings following PCI with major adverse outcomes.^[36,37]^ However, in the present study the difference of bleeding rates in both treatment groups did not affect the development of MACE at 30 days. Not only major but also minor bleedings were once shown to increase mortality.^[3]^ In this study complicated bleeding being revealed to affect an adverse clinical outcome did not differ in both groups and did not influence MACE. Furthermore, no significant differences of TLR or TVR rates were observed in the RC and FC group.

The patency of the radial artery after PCI with TRA was checked by testing the capillary flow distal to the access site while maintaining an occlusive compression of the ulnar artery. And there was no radial artery occlusion in patients undergoing PCI with TRA. In a recent study by Indolfi et al it was demonstrated, that a hand laser perfusion imaging could identify significantly radial artery occlusion in 100% of cases.^[38]^ Indeed, the radial artery occlusion is usually asymptomatic, but not a benign complication. However, diagnosis of post-procedural radial artery occlusion is often missed, also due to demanding diagnostic examination by means of vascular duplex examination. Therefore, the laser perfusion imaging could be considered as an alternative method to check for radial artery occlusion after PCI with TRA in case of suspicion of an occlusion compared to Allen's test or vascular duplex imaging.

## 5 Conclusion

Despite the subsequent use of VCD (StarClose SE) for FC after PCI, TFA was still significantly associated with a higher prevalence of access site bleedings consisting mostly of hematomas compared to TRA and RC using TR Band. The development of advanced vascular closure devices and further clinical research on their use might bear the potential to minimize more efficiently bleedings after PCI with TFA in the upcoming future.

### 5.1 Limitations

This is a nonrandomized study that compares two different PCI accesses. Thus, all limitations of nonrandomized studies are involved, for example, selection bias by individual choices of access sites, sheath diameters, and used techniques and PCI materials. In addition, antithrombotic treatment before and after procedure was not predefined. Due to a higher prevalence of patients with acute coronary syndrome in the FC group, the charge dose of ticagrelor in this group was very different from the RC group. In any case, this difference could also play a role in the development of bleedings during or after PCI. All of these could greatly affect the study results.

A significant association of access-site bleedings after PCI with sheath size was in many previous clinical studies proven. Koeth et al revealed significantly increased bleeding risk in patients undergoing PCI with larger sheath size.^[39]^ However, this could not be statistically proven in our study. Although our multivariable analysis did not show a significant association of sheath diameter with bleedings, we still cannot rule out this plausible correlation.

## Author contributions

**Formal analysis:** Sebastian Baron, Tetyana Shchetynska-Marinova, Melike Tekinsoy.

**Supervision:** Kambis Mashayekhi, Ursula Hoffmann, Martin Borggrefe, Ibrahim Akin.

**Writing – original draft:** Seung-Hyun Kim.

**Writing – review & editing:** Michael Behnes.

Seung-hyun Kim orcid: 0000-0002-1894-7900.
